# Isometric gene tree reconciliation revisited

**DOI:** 10.1186/s13015-017-0108-x

**Published:** 2017-06-13

**Authors:** Broňa Brejová, Askar Gafurov, Dana Pardubská, Michal Sabo, Tomáš Vinař

**Affiliations:** Faculty of Mathematics, Physics, and Informatics, Comenius University, Mlynská dolina, 842 48 Bratislava, Slovakia

**Keywords:** Gene family evolution, Gene tree reconciliation, Level ancestor

## Abstract

**Background:**

Isometric gene tree reconciliation is a gene tree/species tree reconciliation problem where both the gene tree and the species tree include branch lengths, and these branch lengths must be respected by the reconciliation. The problem was introduced by Ma et al. in 2008 in the context of reconstructing evolutionary histories of genomes in the infinite sites model.

**Results:**

In this paper, we show that the original algorithm by Ma et al. is incorrect, and we propose a modified algorithm that addresses the problems that we discovered. We have also improved the running time from $O(N^2)$ to $O(N\log N)$, where *N* is the total number of nodes in the two input trees. Finally, we examine two new variants of the problem: reconciliation of two unrooted trees and scaling of branch lengths of the gene tree during reconciliation of two rooted trees.

**Conclusions:**

We provide several new algorithms for isometric reconciliation of trees. Some questions in this area remain open; most importantly extensions of the problem allowing for imprecise estimates of branch lengths.

## Background

In this paper, we revisit the problem of isometric gene tree reconciliation introduced by Ma et al. [1, 2]. We point out several mistakes in the original publications and provide a corrected and simplified version of the algorithm. We also improve its running time by employing appropriate data structures and solve two new variants of the problem.

**Fig. 1:**
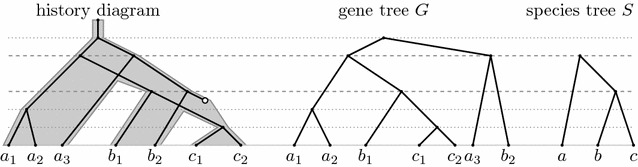
An example of the evolutionary history of a gene family and its corresponding gene tree *G* and species tree *S*. In the history diagram, species are shown as *gray bands*, genes within species as *black lines*, gene losses as *empty circles*. Gene identifiers start with species label; thus $a_1$, $a_2$ and $a_3$ are three copies of the studied gene in species *a*. Duplications are highlighted by *gray dotted horizontal lines*, speciations by *dashed lines*

We will consider evolution of a single gene family. The evolutionary history starts with a single ancestral gene which evolves by a series of duplications, speciations, and losses, resulting in several present-day species, each carrying some number of copies of the studied gene. A particular evolutionary history of a gene family defines gene tree *G* and species tree *S* (see Fig. 1). The leaves of the species tree *S* are the present-day species, and the internal nodes correspond to the speciation events in the history. The leaves of the gene tree *G* are the present-day copies of the gene and the internal nodes correspond to the duplications and the speciations.

Species trees and gene trees can be reconstructed from sequence data by well-established methods [3]. However, one pair of a gene tree and a species tree may correspond to many different histories, because it is not clear, which nodes of the gene tree correspond to speciations in the species tree. The goal of gene tree/species tree reconciliation is to map nodes of the gene tree to the species tree, and thus to reconstruct the evolutionary history of the gene family.

Classical approaches to reconciliation consider only topologies of the gene tree and the species tree. As the reconciliation is not unique, the goal is to find the most parsimonious reconciliation minimizing the number of events. This problem is studied since 1979 [4], and multiple algorithms were developed [5–8].

In this paper, we consider a different variant of the problem called *isometric gene tree reconciliation*. In this problem, branch lengths in both the gene tree and the species tree are known exactly, and the reconciliation should obey them. This problem was introduced by Ma et al. [1], who used this form of gene tree reconciliation as one of the steps in their polynomial-time algorithm to reconstruct evolutionary history of several genomes in a rich model which includes duplications, two and three breakpoint rearrangements, deletions, and insertions under the assumptions of the infinite sites model. This result is rather remarkable, as reconstruction of rearrangement histories is typically NP-hard even in simple models [9].

**Fig. 2:**
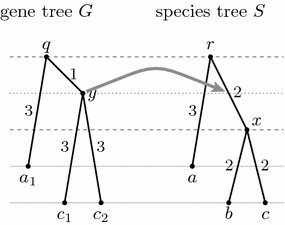
Mapping of node *y* in the isometric reconciliation of rooted gene tree *G* and species tree *S*. A more detailed view of this reconciliation can be found in Fig. 3

If both the gene tree and the species tree are rooted, their isometric reconciliation can be found by a straightforward algorithm. Mapping of leaves is given on input, because we know the species of origin for each gene. To map an internal node *v* of the gene tree, we choose one leaf *u* in its subtree and map *v* to the unique place in the species tree determined by the distance between *u* and *v*. For example, node *y* in Fig. 2 maps to a point in the middle of edge (*r*, *x*), because this is the unique point on the path from the root to leaf *c*, which is situated in distance 3 from *c*. This algorithm is described in more detail as Algorithm 1.

However, Ma et al. [1, 2] consider a more difficult problem, in which the species tree is rooted, but the gene tree is unrooted. This is needed, because in practice most of the phylogenetic reconstruction methods produce unrooted trees. While the species tree can be rooted by including an outgroup, finding an appropriate outgroup for a multi-gene family, which may harbor ancient duplications, is more problematic. Ma et al. give a polynomial-time algorithm for the isometric reconciliation problem, and after some unspecified modifications, apply it to real data with inexact branch lengths. In this paper, we point out several mistakes in their version of the algorithm and provide a corrected version.

We also study two extensions of the problem which were not considered before. First, we extend our algorithm to the case when the species tree is unrooted as well. We also provide an algorithm for the case when both trees are rooted, but the branch lengths of the gene tree are scaled by some unknown factor which needs to be discovered by the algorithm. This is a first step towards a more realistic scenario where the branch length are not known exactly. This particular variant of the problem is motivated by the observation that individual genes often differ in their substitution rates; rate variation is a common component in models of evolution [3].

Reconciliation with some branch length information was also previously studied in several more complex models, such as probabilistic approaches considering branch lengths in *S* [10–12] and models allowing horizontal gene transfer [13, 14].

The rest of the paper is organized as follows. We start by formally defining the problem and describing the simple algorithm for the case when both trees are rooted. Then we briefly describe the algorithm from the work of Ma et al. and point out its problems. Next, we provide a corrected algorithm for reconciling an unrooted gene tree with a rooted species tree. We also describe algorithms for two extensions of the original problem. At the end of the article, we provide further details concerning our evolutionary models; these details are not necessary for the algorithms, but help to interpret their results.

## Preliminaries

In this section, we introduce the notation used in this paper and formally define the problem of isometric reconciliation. Note that Ma et al. define the problem only briefly, and as we will discuss in the next section, their definition is not sufficiently stringent. We will also describe a simple algorithm for isometric reconciliation of two rooted trees.

### Basic tree notation

We will consider rooted and unrooted phylogenetic trees with non-negative branch lengths. Although in this section we briefly consider branches of length zero, all our algorithms assume that the input trees have strictly positive branch lengths. On the other hand, the algorithms do not require that the trees are binary; they can have nodes of higher degree. We might obtain such nodes by contracting branches of length zero in a binary phylogenetic tree. Some of the trees considered in this work also have nodes with only one child; these nodes correspond to subdivisions of edges in a binary tree.

We will now briefly introduce useful tree-related notation. Given two nodes *u* and *v* belonging to the same phylogenetic tree, *d*(*u*, *v*) denotes their *distance*, i.e. the sum of branch lengths on the unique simple path between *u* and *v*. An edge (*u*, *v*) of a rooted or unrooted tree can be considered as an interval of length *d*(*u*, *v*). The point in this interval in distance *d* from node *u* will be denoted as $\text{ pt }(u,v,d)$; this is defined for $0\le d \le d(u,v)$. If a tree is rooted, we will also use notation $\text{ pt }(u,d)$ as a shortcut for $\text{ pt }(u,v,d)$, where *v* is the parent of *u*. We will also use $\text{ pt }(u,d)$ when *u* is the root; it will represent the point on an implicit edge of infinite length leading to the root from above.

We can *subdivide edge* (*u*, *v*) at $\text{ pt }(u,v,d)$ by replacing it with two edges of lengths *d* and *d*(*u*, *v*)−*d* connected to a new node *x*. The inverse operation (i.e. replacing a path of two edges leading through node *x* of degree two by one edge of the same length) will be called *bypassing* node *x*. A *rooted version of an unrooted tree* *T* is created either by choosing one internal node of *T* as the root or by subdividing one of the edges of *T* and selecting the new node as the root.

An *ancestor* of node *v* in a rooted tree is any node on the path from *v* to the root, including *v*. We use the term *proper ancestor* for ancestors excluding *v*. By $\text{ anc }(u,d)$ we denote the point in the tree which is at a distance exactly *d* from *u* on the path towards the root. It can be one of the ancestors of *u*, or if no ancestor is in distance exactly *d*, it is $\text{ pt }(u',d')$ such that $u'$ is an ancestor of *u*, $d'=d-d(u,u')>0$ and for any ancestor $u''$ of $u'$ we have $d-d (u,u'')<0$. Note that we will use this notation only in trees with strictly positive branch lengths, as otherwise there could be multiple ancestors with distance *d*. By $\text{ lca }(u,v)$, we denote the lowest common ancestor of nodes *u* and *v*.

### Isometric mapping and history

Using the introduced notation, we will now define the central object of our study, the isometric mapping between trees.

#### **Definition 1**

An *isometric mapping* from a rooted phylogenetic tree $T_1$ to a rooted phylogenetic tree $T_2$ is a mapping $\Phi$ of nodes of $T_1$ to nodes of $T_2$ such that if node $u\in T_1$ is the parent of *v*, then $\Phi (u)$ is a proper ancestor of $\Phi (v)$ and $d(\Phi (u),\Phi (v))=d(u,v)$; for trees with strictly positive branch lengths this condition is equivalent to $\Phi (u)=\text{ anc }(\Phi (v),d(u,v))$.

Note that by induction, the relationship $\Phi (u)=\text{ anc }(\Phi (v),d(u,v))$ holds for any nodes *u* and *v* in *G* such that *u* is an ancestor of *v*. Given an isometric mapping $\Phi$ and node *v* in $T_2$, by $\Phi ^{-1}(v)$ we denote the (possibly empty) set of nodes of $T_1$ that map to *v*.

**Fig. 3:**
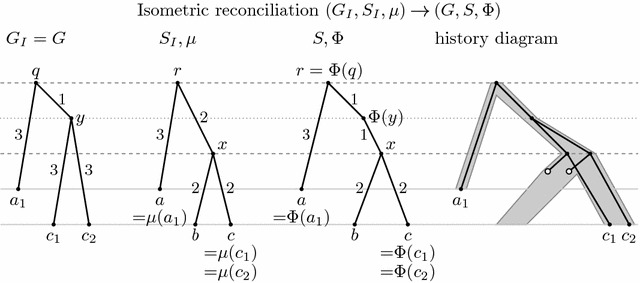
An example of isometric reconciliation. The underlying history, depicted in the diagram on the *right*, consists of two speciations, one duplication and two losses. The two input trees $G_I$ and $S_I$ are shown on the *left*. Note that $G_I$ does not contain the lost genes. Reconciliation results in a new species tree *S*, in which a new node for duplication was added. Isometric mapping $\Phi$ is shown as labels in tree *S*. Input leaf mapping $\mu$ agrees with leaf labels and is shown for completeness as labels in tree $S_I$

Recall that we are interested in studying the evolutionary history of a single gene family consisting of speciations, duplications, and losses. An isometric mapping from the gene tree to the species tree helps us to interpret the nodes of the two trees as these events. A formal definition of a history in our model follows; an example of a history is triple $(G,S,\Phi )$ in Fig. 3.

#### **Definition 2**

*A history* is a triple $(G, S, \Phi )$, where the gene tree *G* and the species tree *S* are rooted phylogenetic trees and $\Phi$ is an isometric mapping from *G* to *S*.

### Inferable histories

Our basic definition of a history is quite general. For example, it allows trees with zero branch lengths. The gene tree may also contain leaves that map to internal nodes of the species tree; such leaves correspond to gene losses. However, input trees for the isometric reconciliation problem are in practice constructed from extant genes, and thus they do not contain any deleted genes. Therefore, our algorithms work with a more restricted set of *inferable histories*. To define them, we first need the following terminology. Consider an arbitrary history $(G, S, \Phi )$. We will call a leaf *v* of *G* *extant* if $\Phi (v)$ is also a leaf in *S* (node *v* then corresponds to an observed gene in a sampled taxon). Node *v* of *G* is called *observable*, if it is an extant leaf or if at least two of its children have at least one extant leaf among their descendants. Non-observable nodes in *G* thus include leaves corresponding to gene losses, internal nodes with only gene loss leaves in their subtrees, but also internal nodes that have extant leaves among descendants, but all such leaves are descendants of a single child. Such a node corresponds to a duplication or a speciation which is not observable because only one copy remains. Note that the input gene trees will contain only observable nodes.

#### **Definition 3**

A history $(G, S, \Phi )$ is called *inferable* if all branch lengths are strictly positive, all nodes of *G* are observable and each node $v\in S$ with exactly one child has $\Phi ^{-1}(v)$ non-empty.

The definition ensures that in an inferable history, all nodes with one child in *S* correspond to duplications. Note that such a node can be the root of the species tree if some duplication happened before the first speciation.

On the other hand, gene trees and species trees in inferable histories may have nodes with more than two children. Such nodes (and even some nodes with two children) correspond to multiple evolutionary events happening simultaneously. A history, in which each internal node of the species tree corresponds to exactly one event, namely a speciation, duplication, or loss, will be called *simple*. A more detailed definition of simple histories as well as the correspondence between simple and inferable histories is described in a separate section at the end of the article.

### Problem specification

Finally, we define the input to our algorithm and the correspondence between this input and the desired output. An example illustrating the following definitions can be seen in Fig. 3.

#### **Definition 4**

An *input partial history* is a triple $(G_I,S_I,\mu )$ such that $G_I$ and $S_I$ are rooted or unrooted phylogenetic trees with positive edge weights, $\mu$ is a mapping from leaves of $G_I$ to leaves of $S_I$, and each internal node *v* in both $G_I$ and $S_I$ satisfies the following:

- If the tree is rooted, *v* has at least two children.
- If the tree is unrooted, *v* has at least three neighbors.

#### **Definition 5**

An inferable history $(G,S,\Phi )$ is called an *isometric reconciliation of the input partial history* $(G_I,S_I,\mu )$ if the following holds.

- Tree *G* is equal to $G_I$ if rooted, or it is a rooted version of $G_I$.
- Tree *S* is obtained from $S_I$ by rooting it (if unrooted), potentially subdividing some edges by new nodes, and potentially adding a path leading to the original root of $S_I$ from above.
- Mapping of every leaf *v* of $G_I$ satisfies $\Phi (v)=\mu (v)$.

### Reconciliation algorithm for rooted trees

If both the gene tree and the species tree are rooted on the input, the reconciliation can be constructed by the following simple algorithm.

#### **Algorithm 1**

Input: partial input history $(G_I,S_I,\mu )$, $G_I$ and $S_I$ rooted.

Output: isometric gene tree reconciliation $(G,S,\Phi )$ of $(G_I,S_I,\mu )$.

We start by mapping nodes of $G=G_I$ to nodes or points on edges of $S_I$. In particular, for every internal node *v* of *G*, we choose some leaf *u* which is a descendant of *v*. The definition of isometric reconciliation then implies that *v* should map to $\text{ anc }(\mu (u),d(u,v))$.

Once all nodes are mapped in this way, we create tree *S* by subdividing edges of $S_I$ and adding a new path from above to the original root so that each node of *G* maps to a node of *S* and not to a point inside an edge. A more detailed description of this process can be found in Algorithm 2.

If the input partial history has an isometric reconciliation, this algorithm is sufficient to find it, because mapping of each node is uniquely determined with respect to the chosen leaf *u*. However, it is possible that the input has no reconciliation, because different choices of leaf *u* can give different mappings of node *v*. Therefore in the final phase of the algorithm, we verify if for each edge (*u*, *v*) of *G* (where *u* is the parent of *v*) we have that $\Phi (u)=\text{ anc }(\Phi (v),d(u,v))$, i.e. that the constructed mapping is a proper isometric mapping. If this condition is violated for any edge, we reject the input as irreconcilable.

Using the techniques which we describe for Algorithm 2, this algorithm can be implemented in $O (N\log N)$ time, where *N* is the total number of nodes in the two input trees.

### Triangulation

The previous simple algorithm maps each internal node *v* based on the distance to a single already mapped descendant. We cannot use this approach for reconciling unrooted gene trees, because we do not know which leaves in the gene tree are descendants of *v*. Instead, the basic ingredient in our algorithm will be a *triangulation*, which maps an internal node based on its distance to two different already mapped nodes. This idea is implicitly used also in the algorithm by Ma et al.; here we state and prove it explicitly.

#### **Definition 6**

Consider a rooted or unrooted tree $G_I$ and a rooted tree $S_I$ with strictly positive branch lengths. Let *x*, *u* and *v* be three (not necessarily distinct) nodes in $G_I$ such that *x* lies on the unique simple path connecting *u* and *v*. Let $u'$ and $v'$ be nodes in $S_I$. By *triangulation* $\Delta (x,u,v,u',v')$ we understand the node or point in $S_I$ constructed as follows:

- Let $$x_u=\text{ anc }(u',d(u,x))$$ and $$x_v = \text{ anc }(v', d(v,x))$$.
- If one of $x_u$ and $x_v$ is a descendant of the other, use the descendant as the triangulation (this includes the case where $x_u=x_v$).
- Otherwise, the triangulation is undefined.

In our algorithms, we will choose nodes *u* and *v* for which $\Phi (u)$ and $\Phi (v)$ have already been found, and we will use $\Delta (x,u,v,\Phi (u), \Phi (v))$ to determine $\Phi (x)$ for another node *x*. The following lemma proves the correctness of such a mapping by triangulation.

#### **Lemma 1**

*Consider a history* $(G,S,\Phi )$ *with strictly positive branch lengths. Let* *x* *be a node on the path connecting nodes* *u* and *v* in *G*. Then $\Phi (x)=\Delta (x,u,v,\Phi (u),\Phi (v))$.

#### *Proof*

Let us consider node $a=\text{ lca }(u,v)$. This node is located on the path connecting *u* and *v* in *G*. There are two possibilities: either *a* is located on the portion of this path between *u* and *x* (including the endpoints), or on the portion between *x* and *v*. These two possibilities are in fact symmetric after switching *u* and *v*. Therefore, we will assume that node *a* is on the path from *u* to *x*. Vertex *u* is then a descendant of *a*, and thus $\Phi (a)=\text{ anc }(\Phi (u),d(u,a))$. Since $d(u,a)\le d(u,x)$, $x_u$ is an ancestor of $\Phi (a)$. On the other hand, *x* is a descendant of *a* and *v* is a descendant of *x*. Therefore, $\Phi (x) = \text{ anc }(\Phi (v),d(v,x))$ and so node $x_v$ will be equal to $\Phi (x)$. Since $x_u$ is an ancestor of $\Phi (a)$ and $x_v$ is a descendant of $\Phi (a)$, $x_v$ is a descendant of $x_u$ and indeed, $x_v=\Phi (x)$ as required. □

## Problems in the original algorithm

The two papers by Ma et al. [1, 2] include the same algorithm for isometric gene tree reconciliation in the case when the input gene tree $G_I$ is unrooted and the species tree $S_I$ is rooted. In this section, we describe some of its details and point out mistakes in the original paper. We start with the original definition of isometric reconciliation as given by Ma et al. (with only slight changes due to different notation and terminology used in this paper). Note that Ma et al. use the same symbol for the input trees $G_I$ and $S_I$ and their output versions *G* and *S*; we have kept their notation for the purpose of the next definition.

### **Definition 7**

(*Original definition*) Any mapping $\Phi$ from a gene tree *G* to a species tree *S* that roots the gene tree is an isometric reconciliation if

1. Every leaf of *G* maps to the leaf of the designated species in *S*.
2. Each internal node of *G* maps to a speciation node in *S* or a point on a branch in *S*.
3. The new root *q* of *G* maps to a point $\Phi (q)$ on a branch in *S* such that any other node *x* in *G* maps to $\Phi (x)$ below $\Phi (q)$ and $d(\Phi (x),\Phi (q)) = d(x,q)$.

**Fig. 4:**
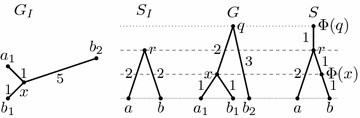
A counter-example for the original definition of isometric reconciliation. Trees $G_I$ and $S_I$ are the input trees, *G* is the rooted version of $G_I$ and *S* is $S_I$ with duplication nodes added. This reconciliation satisfies Definition 7, but it does not correspond to any evolutionary history

Figure 4 shows that this definition is not sufficiently stringent to characterize meaningful reconciliations. The mapping $\Phi$ shown in the figure satisfies all of the above conditions, but does not correspond to any valid history. In particular, node *x* in *G* is a parent of leaf $a_1$, but node $\Phi (x)$ is not an ancestor of $\Phi (a_1)$.

This problem is very easily corrected by demanding that mapping $\Phi$ preserves distances and ancestor relationships between every pair of nodes in *G*, or equivalently, between every pair of adjacent nodes in *G*, as we have done in our Definition 1.

### Overall scheme of the original algorithm

The algorithm of Ma et al. proceeds by first mapping leaves of $G_I$ to corresponding leaves of $S_I$ and then repeatedly choosing one unmapped node *x* from $G_I$ which has at least two of its three neighbours already mapped. Each such node *x* is mapped to its corresponding point $\Phi (x)$, and if one of the edges incident to *x* contains the correct position of the root, the gene tree is rooted. This process continues, until only one node remains.

The overall scheme of the algorithm reveals another minor issue: the algorithm does not work for gene trees with two leaves. The leaves can be mapped trivially, but we also need to find the position of the root on the edge connecting them in $G_I$, and since in the algorithm, rooting is done simultaneously with mapping internal nodes, it is not obvious how to find the root in this case.

### Mapping one node

**Fig. 5:**
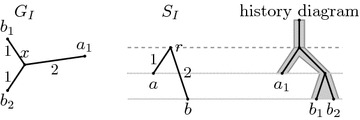
An example which can be reconciled, but which the original algorithm may recognize as irreconcilable. When mapping node *x*, all three neighbors are already mapped, and thus the algorithm by Ma et al. can choose any two of them as nodes *u* and *v*. If it chooses $b_1$ and $b_2$, it works correctly, but if it chooses $a_1$ and $b_1$, it will reject the tree in step 7(b)iii of the original algorithm [1, page 17 of the Supplement]

The algorithm for mapping an internal node and, if appropriate, rooting the gene tree, consists of a rather extensive case analysis, with about ten different cases. After simulating the algorithm on several examples, we have discovered that it does not always work correctly. Figure 5 shows a simple input, which can be reconciled. The algorithm maps the only internal node *x* correctly, but sometimes fails during rooting, rejecting the input as irreconcilable. When mapping the last internal vertex, the algorithm arbitrarily chooses, which two neighbours of this vertex are considered first, and depending on this choice, the algorithm may fail or succeed on this input.

### Speciation and duplication happening at the same time

**Fig. 6:**
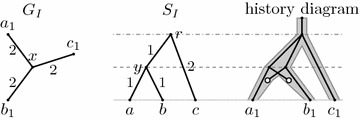
An input rejected by the original algorithm where the reconciliation maps a duplication and a speciation to the same point in the history

Ma et al. assume that the input trees are binary and that two events (two duplications or a duplication and a speciation) never happen at the same time. However, even binary input trees may lead to situations, where two events happen at the same time. A simple example is when the root of *G* coincides with one of the internal vertices of $G_I$, and thus it has three children. This situation is handled by the original algorithm and rejected in case 7(b)iii. An example of such an input is shown in Fig. 6.

**Fig. 7:**
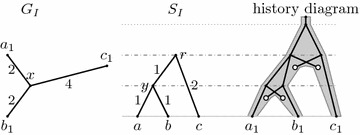
An input accepted by the original algorithm, where reconciliation maps duplication and speciation to the same point in the history

Figure 7 shows a similar input, with only a single branch length changed. Here also a duplication happens at the same time as a speciation, but the rooted gene tree *G* remains binary thanks to later losses. The original algorithm accepts this input, which seems inconsistent with handling the input from Fig. 6. Note that both of these inputs can be reconciled so that they satisfy our definition of isometric reconciliation (as well as the weaker original definition).

Our algorithms work under a relaxed evolutionary model, in which we allow an arbitrary combination of events to happen at the same time. Thus our modified algorithm shown in the next section will reconcile both of these inputs.

If instead we wish to stipulate that no two events may happen at the same time, we need to modify the algorithm so that it rejects both of these inputs and also modify the definition of isometric reconciliation so that it allows only histories satisfying this requirement. At the end of this article, we discuss simple histories in which each node of the species tree corresponds to a single event. The strict definition of isometric reconciliation could require that the output history is an inferable version of some simple history with strictly positive branch lengths (see Definition 10). Note that this condition can be verified for a given inferable history in a postprocessing step of a reconciliation algorithm using the characterization given in Claim 4 and Definition 8.

### Summary of issues

To summarize, the original definition of isometric reconciliation allows nonsense mappings that do not correspond to any evolutionary history and does not adequately handle cases with simultaneous duplication and speciation. In addition, the original algorithm does not handle gene trees with two leaves and sometimes fails to root valid inputs. In the next section, we present a new algorithm that corrects these problems and, at the same time, simplifies the proof of correctness by reducing the case analysis to a minimum.

## The modified algorithm

In this section, we describe a new version of the algorithm for finding isometric gene tree reconciliation $(G,S,\Phi )$ satisfying Definition 5, provided that in the input partial history $(G_I,S_I,\mu )$, gene tree $G_I$ is unrooted and species tree $S_I$ is rooted. Although the overall idea is similar to the original algorithm, we have made it more modular, with several passes through the tree, each relatively simple. This allows us to avoid complicated case analysis in both the algorithm and the proof.

To keep the algorithm efficient, we will defer explicit construction of *S* with added duplication nodes. In the first stages of the algorithm we create mapping $\Phi$, but when a node of $G_I$ maps to $\text{ pt }(u,d)$, we will keep the mapping simply as a pair (*u*, *d*).

### **Algorithm 2**

Input: partial input history $(G_I,S_I,\mu )$, $G_I$ unrooted, $S_I$ rooted.

Output: isometric gene tree reconciliation $(G,S,\Phi )$ of $(G_I,S_I,\mu )$.

**Fig. 8:**
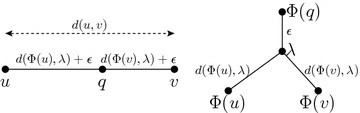
Illustration of stage 3: creating a potential root *q* inside edge (*u*, *v*)

- **Stage 1:** Initialization.
  Set $G=G_I$ and for each leaf *v* of *G*, set $\Phi (v)=\mu (v)$.
- **Stage 2:** Map internal vertices of *G*.
  Repeatedly consider an unmapped internal node *x* of *G* with at least two mapped neighbors *u* and *v*. Set $\Phi (x)=\Delta (x,u,v,\Phi (u),\Phi (v))$. If this triangulation is not defined, reject the input.
- **Stage 3:** Add new nodes to *G*.
  Consider each edge (*u*, *v*) of *G*. We want to decide if this edge should be subdivided by a new node, which will be a *potential root.* Let $\lambda =\text{ lca }(\Phi (u),\Phi (v))$ and let $\epsilon = (d(u,v)-d(\Phi (u),\lambda )-d(\Phi (v),\lambda ))/2$. If $\epsilon >0$ or $\lambda \notin \{\Phi (u),\Phi (v)\}$, create a new node $q=\text{ pt }(u,v,d(\Phi (u),\lambda )+\epsilon )$ (see Fig. 8). Set $\Phi (q)=\text{ anc }(\lambda ,\epsilon )$.
- **Stage 4:** Create *S*.
  For each node *u* of $S_I$ create a list of implicit nodes of the form $\text{ pt }(u,d)$ which were created in Stages 2 and 3 as $\Phi (x)$ for some node *x* of *G*. This can be done by a single traversal through all nodes of *G*. For each node *u* of $S_I$, sort these implicit nodes by distance *d* and remove duplicates. Then replace the edge from *u* to its parent by a new path leading through all nodes of the form (*u*, *d*) in the sorted order.
- **Stage 5:** Root *G*.
  After Stage 3, the root should be among nodes of *G*. For each node *v* of *S* create set $\Phi ^{-1}(v)$ by traversing all nodes of *G* and inserting each into the corresponding set. Select a node *u* in *S* such that $\Phi ^{-1}(u)$ is non-empty and each proper ancestor *v* of *u* has $\Phi ^{-1}(v)$ empty (there is always such a node *u* for non-empty *G*). Select one of the nodes in $\Phi ^{-1}(u)$ as the root.
- **Stage 6:** Check that mapping $\Phi$ is isometric.
  Consider each edge (*u*, *v*) of *G* where *u* is the parent of *v* and check that it satisfies condition $\Phi (u)=\text{ anc }(\Phi (v),d(u,v))$ from Definition 1.

### Proof of correctness

First let us assume that input partial history $(G_I, S_I, \mu )$ has some isometric reconciliation $(G^*,S^*,\Phi ^*)$. We will prove that the algorithm will indeed output $(G^*,S^*,\Phi ^*)$ on this input, regardless of arbitrary choices in the algorithm, such as the order of processing of vertices in Stage 2. This implies that the isometric reconciliation is unique, because if there were two distinct reconciliations, the algorithm cannot produce both of them simultaneously.

Correctness of the mapping constructed in Stage 2 can be obtained by induction from the properties of triangulation. If for both mapped neighbors of *x* we have $\Phi (u)=\Phi ^*(u)$ and $\Phi (v)=\Phi ^*(v)$, then by Lemma 1 we have that $\Phi (x)=\Phi ^*(x)$. Since the mapping of leaves is given on the input, the algorithm will correctly recover $\Phi ^*$ for each internal node of $G_I$. Note that the existence of an unmapped node with at least two mapped neighbors in each iteration of the algorithm is guaranteed by basic properties of trees.

The root of $G^*$ is either one of the nodes of $G_I$ or a new node subdividing some edge; this is the only allowed addition of a node to *G*. We will now prove that the only node potentially added in Stage 3 is the root of $G^*$.

#### **Lemma 2**

*If Stage 3 of the algorithm considers an edge* (*u*, *v*)* of* $G_I$ *with its endpoints correctly mapped to* *S*,* it will subdivide this edge by a new node if and only if* $G^*$ *has a root inside this edge. The new node will be created at the correct position and mapped correctly.*

#### *Proof*

Let $q^*$ be the root of $G^*$, and let us consider three cases regarding the position of $q^*$ with respect to *u* and *v*.

*Case 1* $q^*$ is inside edge (*u*, *v*), excluding the endpoints. Let $d=d(u,v)$ and let $\delta =d(q^*,u)$. We have $0<\delta <d$. Since $q^*$ is the parent of both *u* and *v* in $G^*$, $\Phi ^*(q^*)=\text{ anc }(\Phi ^*(u),\delta )= \text{ anc }(\Phi ^*(v),d-\delta )$. We will consider two subcases concerning the position of $\lambda =\text{ lca }(\Phi ^*(u),\Phi ^*(v))$.

- If $\lambda =\Phi ^*(q^*)$, a new node will be created, because $\lambda \notin \{\Phi (u),\Phi (v)\}$. We have $\epsilon =(d-\delta -(d-\delta ))/2 = 0$, so the node will be created at distance $\delta$ from *u*, as desired.
- If $\Phi ^*(q^*)$ is not $\lambda$, $\lambda$ must be a descendant of $\Phi ^*(q^*)$, with $d(\lambda ,\Phi ^*(q^*))=\epsilon '>0$. Note that in this case, it is possible that $\lambda \in \{\Phi ^*(u),\Phi ^*(v)\}$. However, $\delta =d(\Phi ^*(u),\lambda )+\epsilon '$ and $d-\delta =d(\Phi ^*(v),\lambda )+\epsilon '$ and thus $\epsilon =(d-(\delta -\epsilon ')-(d-\delta -\epsilon '))/2=\epsilon '>0$. Thus a node will be created, and its distance from *u* will be correctly set to $d(\Phi ^*(u),\lambda )+\epsilon =\delta$.

*Case 2* After removal of edge (*u*, *v*) from $G^*$, $q^*$ is in the connected component containing *u*, including the case $q^*=u$. Then $\Phi ^*(u)=\text{ anc }(\Phi ^*(v),d(u,v))$ and thus $\lambda =\Phi ^*(u)$. In addition, $d(\Phi ^*(u),\lambda )=0$, $d(\Phi ^*(v),\lambda )=d(u,v)$ and thus $\epsilon =0$. No node will be created, which is correct, as in this case the root is not inside this edge.

*Case 3* $q^*$ is in the connected component containing *v*. This case is symmetrical to case 2. □

After Stage 3, we will thus have all nodes of $G_I$ correctly mapped to *S*, and if the root of *G* is not one of the nodes of $G_I$, it was also correctly added and mapped. Stage 4 simply changes the representation of *S* from implicit to explicit. The definition of isometric mapping implies that if $q^*$ is the root of $G^*$, then all other nodes of $G^*$ map to proper descendants of $\Phi ^*(q^*)$ and thus $q^*$ is the only node that can be selected in Stage 5 as the root. Thus the algorithm will correctly recover the correct answer $(G^*, S^*, \Phi ^*)$.

To finish the proof of correctness, we need to prove that the algorithm will correctly reject any input for which no reconciliation exists. Thanks to explicit checks in Stage 6, the output will be always a correct isometric reconciliation, with one possible exception: Stage 3 may possibly subdivide edges of *G* by nodes which are not the root. However if a triple $(G,S,\Phi )$ exists which satisfies all conditions of isometric reconciliation except for some spurious edge subdivisions in *G*, a proper isometric reconciliation would exists as well. This is a contradiction, because in this part of the proof, we assume that the input cannot be reconciled.

### Running time analysis

Let *m* be the number of nodes of $G_I$ and *n* the number of nodes of $S_I$. Ma et al. claim that their algorithm works in *O*(*mn*) time. We will prove that a more efficient implementation of isometric reconciliation is possible. Within the algorithm, we use several nontrivial operations on *S*:

- Finding $\text{ lca }$ of two nodes. We can use efficient data structures for solving $\text{ lca }$ queries in *O*(1) time after *O*(*n*) preprocessing of the tree [15, 16].
- Determining if node *u* is an ancestor of *v*. This is equivalent to asking if $u=\text{ lca }(u,v)$.
- Computing the distance between node *v* and its ancestor *u*. This can be done in *O*(1) time by keeping the distance from the root of $S_I$ in each node and subtracting these distances for *u* and *v*.
- Finding $\text{ anc }(u,d)$. This operation is known as level ancestor. For unweighted trees, it can be solved in *O*(1) time after *O*(*n*) preprocessing [17] and for trees with integer weights in $O (\log \log u)$ time, where *u* is the maximum edge weight [18]. Below, we outline a simplified version of this data structure which achieves $O (\log n)$ time per query, but works for arbitrary edge weights, as edge weights in phylogenetics are typically not expressed as integers.

Using these building blocks, the rest of the algorithm is relatively straightforward. For Stage 2, we maintain a counter of mapped neighbors for each internal node of $G_I$ and a stack of unprocessed nodes with at least two neighbors already mapped. In each step, we remove one node from the stack, map it, and increase the counters of its neighbors. If any counter reaches 2, the corresponding node is added to the stack. The overall overhead for selecting nodes for mapping in Stage 2 is thus *O*(*m*), and mapping each node works in $O (\log n)$. Stage 3 involves a simple loop through all edges, and each edge is processed in $O (\log n)$ time. Stage 4 works in $O (n + m\log m)$ time and Stages 5 and 6 work in $O (n+m)$ time. The overall running time of the algorithm is thus $O (N\log N)$, where $N=n+m$.

Linear-time precomputation for various tree operations can be initially done for the original tree $S_I$ and after Stage 4 recomputed for *S*. However, in Stages 2 and 3, some queries will use as arguments implicit nodes $\text{ pt }(u,d)$ instead of regular nodes of $S_I$. All tree queries can be easily extended to work also for such generalized arguments. For example, $\text{ anc }(\text{ pt }(u,d_1),d_2)=\text{ anc }(u,d_1+d_2)$. When computing $\text{ lca }(\text{ pt }(u,d_1),\text{ pt }(v,d_2))$, we can compute $\lambda =\text{ lca }(u,v)$ and then distinguish several cases based on whether $\lambda \in \{u,v\}$.

### A simple level ancestor for arbitrary weights

For completeness, we briefly describe a data structure for finding $\text{ anc }(u,d)$ in $O(\log n)$ time for arbitrary edge weights, provided that we can do addition, subtraction, and sign operation in constant time. We use a simplified version of the data structure by Amir et al. [18]; the simplification is possible thanks to the fact that the running time is worse than the running time achievable for integer edge weights.

Let node weight *w*(*u*) be the sum of edge weights on the path from the root to node *u*. To compute $\text{ anc }(u,d)$, we are looking for the highest ancestor *v* of *u* such that $w(u)-w(v)\le d$. If we had only a single path instead of a tree, we would be looking for a predecessor of value $x=w(u)-d$ in the sequence of node weights. Since this sequence is increasing, we can use binary search to find the desired index *v*.

In a general tree, we will use the heavy path decomposition [15]. An edge connecting node *v* to its parent *p* in a tree is called *heavy* if the size of the subtree rooted at *v* (the number of nodes) is at least half of the size of the subtree rooted at *p*. Otherwise the edge is called light. For each node, at most one of its children is connected to it by a heavy edge. Therefore, heavy edges form a set of vertex-disjoint paths. Vertices which are not incident to any heavy edge will be considered as heavy paths of length 0 so that each node is included in exactly one heavy path.

We create an array of node weights for each heavy path. Each vertex *v* also keeps the reference to the highest node on its heavy path. When searching for $\text{ anc }(u,d)$, we search along the path from *u* to the root to find the heavy path that contains the answer. Thanks to the properties of the heavy path decomposition, there are at most $O (\log n)$ light edges on any leaf-to-root path, and thus we can use linear search to iterate through heavy paths encountered on the way to the root. In constant time, we can jump to the head of the path and comparing *x* to the value stored in the head and in the head’s parent, we can determine if this path contains the answer. Within the correct path, we then find the answer by binary search. The overall time is thus $O (\log n)$.

The data structure for integer weights by Amir et al. [18] uses binary search over heavy paths, which requires repeated use of the unweighted level ancestor data structure. Instead of binary search within a path, they use efficient data structures for the predecessor problem with integer keys.

## Extensions of the problem

In this section, we consider two extensions of the isometric reconciliation problem. First, we consider the situation when both input trees are unrooted. Note that if the input species tree is rooted, there is at most one reconciliation, even if $G_I$ was unrooted. This is no longer the case when both $S_I$ and $G_I$ are unrooted. Next we will return to the simple scenario when both input trees are rooted, but allow the branch lengths of $G_I$ to be scaled by an arbitrary positive scaling factor.

### Unrooted input trees

**Fig. 9:**
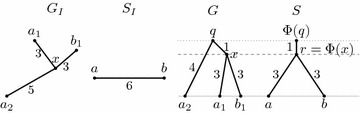
An example of unrooted input trees $G_I$ and $S_I$ which have only one possible isometric reconciliation $(G,S,\Phi )$

**Fig. 10:**
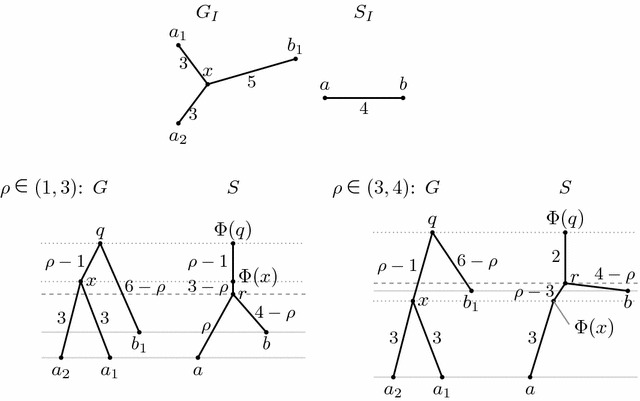
An example of unrooted input trees $G_I$ and $S_I$ such that the species tree can be rooted at any point $\text{ pt }(a,b,\rho )$ for $\rho \in [1,4)$. The *lower part* of the figure shows separately solutions for $\rho \in (1,3)$ and $\rho \in (3,4)$. These two cases differ in the position of $\Phi (x)$. For $\rho =1$, node *q* coincides with *x* (and thus $\Phi (q)$ coincides with $\Phi (x)$) and for $\rho =3$, node *r* coincides with $\Phi (x)$. Case $\rho =4$ is not possible because our definitions do not allow rooting a phylogenetic tree in its leaf. Note that the species tree cannot be rooted at position $\text{ pt }(a,b,\rho )$ for $\rho \in (0,1)$

If both input trees are unrooted, some inputs may have multiple reconciliations. For example, the input in Fig. 9 has a unique reconciliation, while in a similar input in Fig. 10, the species tree can be rooted at any point $\text{ pt }(a,b,\rho )$ for $\rho \in [1,4)$ inside its only edge (*a*, *b*).

We will now describe a different version of the algorithm for unrooted $G_I$ and rooted $S_I$, which will form the basis of our algorithm for unrooted $S_I$.

#### **Algorithm 3**

Input: partial input history $(G_I,S_I,\mu )$, $G_I$ unrooted, $S_I$ rooted.

Output: isometric gene tree reconciliation $(G,S,\Phi )$ of $(G_I,S_I,\mu )$.

In the first part of this algorithm, we consider each edge (*u*, *v*) of $G_I$ separately, and run modified Stages 2, 3, and a part of Stage 6 in the following three steps.

- **Step A:** For each $x\in \{u,v\}$, map *x* as follows. If *x* is a leaf, set $\Phi (x)=\mu (x)$. Otherwise let $\ell _1$ and $\ell _2$ be two leaves of *G* such that *x* is on the simple path connecting them. Set $\Phi (x)=\Delta (x, \ell _1, \ell _2, \mu (\ell _1), \mu (\ell _2))$. If the triangulation is not defined, reject the input.
- **Step B:** If needed, subdivide edge (*u*, *v*) by a new potential root *q*, as in Stage 3 of Algorithm 2.
- **Step C:** If the edge was not subdivided, check that $d(\Phi (u),\Phi (v))=d (u,v)$. If the edge was subdivided by *q*, do an analogous distance check for both new edges (*u*, *q*), (*q*, *v*). If the check fails, reject the input.

Step A is a modified version of Stage 2. Originally we mapped nodes by triangulating from already mapped neighbors of a node; now we use only known mapping for leaves of *G*. This change allows individual edges to be processed independently from each other. Correctness of the mapping is guaranteed by Lemma 1. However, internal nodes belong to multiple edges, and thus they will be mapped multiple times in Step A. To keep the mapping consistent, we will deterministically choose the same pair of leaves $\ell _1$ and $\ell _2$ each time when mapping node *x*. Once this process is completed for all edges, we will run the original Stages 4, 5 and 6 to finish creating $S_I$, $G_I$ and to check the correctness of the mapping.

We will now describe the algorithm for reconciling two unrooted trees.

#### **Algorithm 4**

Input: partial input history $(G_I,S_I,\mu )$, $G_I$ and $S_I$ unrooted.

Output: the set of all isometric gene tree reconciliations $(G,S,\Phi )$ of $(G_I,S_I,\mu )$.

This algorithm is more complex, and we will describe it on the following pages. It is based on simulating Algorithm 3 on multiple inputs with different root positions in $S_I$. The problem with applying the above algorithm for unrooted species tree is that the results of many intermediate operations (namely computing $\text{ anc }$ and $\text{ lca }$, determining descendant-ancestor relationships, computing $\epsilon$ when adding a root, and computing distances) are dependent on the position of the root of the species tree. However, when we move the root only by a small distance, the results of these operations often remain the same or also change only by a small amount. The idea of our algorithm is to split all possible positions of the root into intervals that will produce essentially the same results.

*Unequivocal intervals and the outline of the algorithm.*

Consider edge $(x_a,x_b)$ of $S_I$ and interval (*a*, *b*) such that $0\le a\le b\le d (x_a,x_b)$. Consider rooted versions of $S_I$ with root $r=\text{ pt }(x_a,x_b,\rho )$ for $\rho \in (a,b)$, i.e., the root *r* is inside interval (*a*, *b*) at a distance $\rho$ from $x_a$. We will say that the interval (*a*, *b*) is *unequivocal* if for any two $\rho _1$ and $\rho _2$ from (*a*, *b*), Algorithm 3 will produce essentially the same reconciliation, possibly differing only in branch lengths, and also all intermediate steps in the algorithm will give results which differ only in distances and do not alter the decisions of the algorithm in an important way. We will leave this requirement vague at the moment; we will give further details when discussing particular steps of the algorithm.

As we will show, we can simulate Algorithm 3 for all points in an unequivocal interval simultaneously. However, since the exact position of the root is unknown, we are not able to uniquely determine exact locations of some points which are the result of operations such as $\text{ anc }(x,d)$, particularly for points located above the root. We will express their positions parametrically as $\text{ pt }(r,d+c\rho )$, where parameter $\rho$ is the unknown distance of the root from $x_a$, and *d* and *c* are known constants. We will call points of this form *parametric*, while the uniquely placed points will be *fixed*.

Our algorithm also needs to discover unequivocal intervals. This will proceed as follows. We start with a whole edge $(x_a,x_b)$ as a candidate unequivocal interval, i.e. $a=0$ and $b=d(x_a,x_b)$. We will simulate Algorithm 3, attempting to either produce a single parametric reconciliation for this interval, or to reject the whole interval as a possible root location. However, in the process we may discover that some decision in the algorithm cannot be done uniformly for the whole interval, and thus the interval will be split into two subintervals. These subintervals become new candidate unequivocal intervals and the algorithm will continue processing each of them separately. Thus we start with one interval per edge and successively divide intervals into smaller and smaller parts, until we obtain intervals which are unequivocal. The algorithm will produce an answer for each such unequivocal interval.

Note that the simulation only deals with points inside open intervals (*a*, *b*). In addition, we separately run Algorithm 2 or 3 for $S_I$ rooted in every internal node and for $S_I$ rooted at each unequivocal interval boundary produced by the algorithm.

*Simulating the algorithm for a single candidate unequivocal interval.*

Consider now root positions inside a single interval (*a*, *b*) on edge $(x_a,x_b)$. We will now describe how to produce a parametric reconciliation by simulating Algorithm 3. If the interval (*a*, *b*) is not unequivocal, the simulation will detect it and split the interval into subintervals at value of $\rho$ for which some intermediate result of the algorithm materially changes.

Consider now running Steps A, B and C of Algorithm 3 on some edge (*u*, *v*) of *G*. In Step A, we start by computing $x_u$ and $x_v$ needed in triangulation (see Definition 6). Both of these nodes are found as $\text{ anc }(\ell ,d)$ for some leaf $\ell$ and distance *d*. Since $S_I$ is rooted inside edge $(x_a, x_b)$, leaf $\ell$ must be a descendant of $x_a$ or $x_b$. First let us assume that it is a descendant of $x_a$.

We need to consider three cases. First, if $d\le d(\ell ,x_a)+a$, the position of $\text{ anc }(\ell ,d)$ is fixed: it is somewhere in the subtree rooted at $x_a$ or inside edge $(x_a,r)$ of *S* in a known distance from $x_a$. Second, if $d\ge d(\ell ,x_a)+b$, point $\text{ anc }(\ell ,d)$ will be parametric at $\text{ pt }(r,d-d(\ell ,x_a)-\rho )$. And finally, if $d-d(\ell ,x_a)\in (a,b)$, point $\text{ anc }(\ell ,d)$ may be either inside edge $(x_a,r)$ of *S* or above the root *r*, depending on the exact position of the root, which means that interval (*a*, *b*) is not unequivocal. We will subdivide interval (*a*, *b*) at value $d-d(\ell ,x_a)$, where the relative position of $\text{ anc }(\ell ,d)$ and *r* changes. In each of the two new intervals, we will have $\text{ anc }(\ell ,d)$ either fixed or parametric as above, and thus this particular step of the algorithm will run without producing any new subintervals.

If $\ell$ is a descendant of $x_b$, the situation is similar. For simplicity, let $\rho '$, $a'$ and $b'$ be distances from $x_b$ analogous to $\rho$, *a*, *b*, i.e. $\rho '=d(x_a,x_b)-\rho$, $a'=d(x_a,x_b)-a$, and $b'=d(x_a,x_b)-b$. Then if $d\le d(\ell ,x_b)+b'$, point $\text{ anc }(\ell ,d)$ is fixed; if $d\ge d(\ell ,x_b)+a'$, we get a parametric form $\text{ anc }(\ell ,d) = \text{ pt }(r,d-d(\ell ,x_b)-\rho ') = \text{ pt }(r,d-d(\ell ,x_b)-d(x_a,x_b)+\rho )$, and if $d-d(\ell ,x_b)\in (b',a')$, we split interval (*a*, *b*) at value $d(x_a,x_b)-d+d(\ell ,x_b)$.

After computing both $x_u$ and $x_v$ in this way, the original interval (*a*, *b*) was split into up to three smaller intervals, each of which is processed separately. Points $x_u$ and $x_v$ might be in a given interval either fixed or parametric of the form $\text{ pt }(r,d+c\rho )$, where $c\in \{-1,1\}$. To conclude triangulation, we need to determine if one of $x_u$ and $x_v$ is a descendant of the other. This can be done easily if at least one of them is fixed or if both of them are parametric with the same value of *c*. However, if $x_u=\text{ pt }(r,d_1+c_1\rho )$, $x_v=\text{ pt }(r,d_2+c_2\rho )$ and $c_1\ne c_2$, we need to solve the linear equation $d_1+c_1\rho = d_2+c_2\rho$. If the solution $\rho$ of this equation is inside (*a*, *b*), we split the interval at this value of $\rho$. Each of the new subintervals (or the whole interval if there was no split) then has the same descendant-ancestor relationship between $x_u$ and $x_v$ across its whole length. Overall, the algorithm either rejects the current interval as a possible location of the root or selects one of $x_u$ and $x_v$ as $\Phi (x)$.

The mapping of one node thus can produce up to 5 new interval endpoints, and since we map both *u* and *v*, we get at most 10 endpoints and 11 subintervals in total. In Step B (Stage 3 of Algorithm 2), we start by computing $\lambda =\text{ lca }(\Phi (u),\Phi (v))$. Again, if at least one of $\Phi (u)$ and $\Phi (v)$ is fixed or if they are both parametric with the same *c*, $\lambda$ can be easily computed. Conversely if they are both parametric with different values of *c*, we solve the linear equation, split the interval as needed, and determine which is the ancestor of the other in each resulting interval.

Next we need to compute $\epsilon = (d(u,v)-d(\Phi (u),\lambda )-d(\Phi (v),\lambda ))/2$. For this, we need to compute $d(\Phi (u),\lambda )$ and $d(\Phi (v),\lambda )$. Each of these two distances is measured between two points which may be either fixed or parametric, and thus the distance is in the form $d+c\rho$, where $c\in \{0,1,-1,2,-2\}$, and thus $\epsilon$ is also of such a form with $c\in \{0,0.5,-0.5,1,-1,1.5,-1.5,2,-2\}$. To determine, if $\epsilon$ is positive, we solve the equation $d+c\rho =0$ and split the current interval if the solution is inside it. Note that the position of the new root *x* in *G* might also be parametric of the form $\text{ pt }(u,v,d+c\rho )$ and $\Phi (x)=\text{ anc }(\lambda ,\epsilon )$ might be parametric with *c* from some finite set of values.

Finally, in Step C we compare distances between adjacent nodes *p* and *q* in *G* and $\Phi (p)$ and $\Phi (q)$ in *S*; this is done for one or two pairs of nodes depending on whether edge (*u*, *v*) was subdivided. The distances in both trees might be parametric and to establish equality, we may again need to solve a linear equation and split the interval. In addition, to measure distance between two parametric points in *S* of the form $\text{ pt }(r,d_1+c_1\rho )$ and $\text{ pt }(r,d_2+c_2\rho )$, we need to distinguish, which of them is the ancestor of the other, and thus we may need to split the current interval at the point where $d_1+c_1\rho =d_2+c_2\rho$.

Overall, in processing one edge (*u*, *v*) of $G_I$, we have split the original candidate unequivocal interval covering the whole edge $(x_a,x_b)$ into *O*(1) subintervals and for each we have executed Steps A, B and C. Some of these subintervals might have been rejected by the algorithm. This is done independently for every edge $(x_a, x_b)$ of $S_I$ and every edge (*u*, *v*) of $G_I$, and thus overall we may have up to *O*(*nm*) interval endpoints. We will create a new set of candidate unequivocal intervals by pooling all interval endpoints together and creating a candidate interval between every two adjacent endpoints. Within such a new candidate unequivocal interval, the completed stages of the algorithm would run without producing further endpoints.

For each of these intervals we now consider the remaining stages of the algorithm. Let us first assume that we do not need an explicit representation of *S* and let us skip Stage 4. In Stage 5, we create lists $\Phi ^{-1}(x)$ for both explicit and implicit nodes of *S*; note that implicit nodes might be fixed or parametric. Let us consider the set *R* of nodes for which $\Phi ^{-1}(x)$ is non-empty. Among nodes of *R* which are parametric with the same value of *c*, we can eliminate all except the one with the highest value of *d*, which is the only potential candidate for the root. Also any parametric node eliminates all fixed nodes, which are necessarily its descendants. If we are left with several candidate parametric nodes with different values of *c*, we can perform all pairwise comparisons by solving linear equations and subdividing the current interval further until each new interval has a uniquely determined highest value. Since *c* can have at most *O*(1) different values, we will subdivide each interval *O*(1) times, thus keeping the number of intervals *O*(*nm*).

In Stage 6, we need to compare distances and ancestor relationships between adjacent nodes *u* and *v* of *G* and their counterparts $\Phi (u)$ and $\Phi (v)$ of *S*. However, since we have already done a partial correctness check in Step C, no further interval subdivision is necessary in this step.

If desired, it is also possible to explicitly construct *S*. For that we need to order all the parametric nodes by the size of their value $d+c\rho$ so that they can be placed on the new path leading to the root. Again, implicit nodes with the same *c* can be ordered easily by their value of *d*. To compare other pairs, we solve all pairwise equations of the form $d_1+c_1\rho =d_2+c_2\rho$ and subdivide the current interval at the points where this equation has a solution. In each resulting interval, the relative order of all implicit nodes is uniquely determined. For each starting interval, we solve $O (m^2)$ equations, obtaining $O (m^2)$ intervals. Thus the overall number of intervals is $O (nm^3)$. The running time is $O (nm^3 (n+m) \log (n+m))$ due to data structures needed for level ancestor.

To summarize, the algorithm described above produces a solution which is a set of intervals on the edges of $S_I$. For each interval we obtain a parametric reconciliation in which the position of the nodes above the root is given in the form $\text{ pt }(r,d+c\rho )$ where $\rho$ is the distance of the root position from some node $x_a$ of $S_I$. Our algorithm runs in $O (nm^3 (n+m)\log (n+m))$ time. The main factor is the number of intervals which we estimated as $O (nm^3)$, but perhaps a better upper bound on the number of intervals can be found.

### Scaling branch lengths

So far we have assumed that the branch lengths of the two input trees are known exactly. In this section, we consider a slight relaxation of this assumption, in which we assume that all branch lengths in the gene tree should be multiplied by some unknown constant factor $\alpha >0$. For tree *G*, let $\alpha G$ denote the tree obtained from *G* by multiplying all branch lengths by $\alpha$. The question is for which values of $\alpha$ is gene tree $\alpha G_I$ reconcilable with $S_I$?

This variant of the problem is an idealized model of the situation where different genes evolve by different rates. We will address this problem for the simplest scenario where both input trees $G_I$ and $S_I$ are rooted. We will also assume that for each leaf *v* of $S_I$, $\mu ^{-1}(v)$ is non-empty. This condition can be easily ensured by input preprocessing, where we delete all subtrees of $S_I$ to which no leaf of $G_I$ maps.

We say that a rooted tree is *ultrametric*, if all its leaves are at the same distance from the root [3]. Clearly, if the input trees can be reconciled and one of them is ultrametric, the other must be ultrametric as well; otherwise distances from the root to some leaves could not match in the mapping. It turns out that ultrametric and non-ultrametric trees behave quite differently. The following two claims state the results, and their proofs include algorithms for finding appropriate values of $\alpha$.

#### **Claim 1**

*If neither* $G_I$ *nor* $S_I$ *are ultrametric, there is at most one value of* $\alpha >0$ *such that* $\alpha G_I$ *can be reconciled with* $S_I$.

#### *Proof*

Let *q* be the root of $G_I$ and let *a* and *b* be two leaves of $G_I$ such that $\delta _G = d(q,a)- d(q,b)$ is greater than zero. Such leaves must exist in a tree which is not ultrametric. Let *r* be the root of $S_I$, and let $\delta _S = d(r,\mu (a))-d(r,\mu (b))$. Clearly, we need to scale $G_I$ so that $\alpha \delta _G=\delta _S$, which is possible for at most one value of $\alpha >0$.

Once we have $\alpha$ fixed, we can run Algorithm 1 for rooted $G_I$ and $S_I$ to check if $\alpha G_I$ and $S_I$ are reconcilable. □

#### **Claim 2**

*If both* $G_I$ *and* $S_I$ *are ultrametric, then* $\alpha G_I$ *and* $S_I$ *can be reconciled for all* $\alpha \ge \alpha ^*$ *for some value* $\alpha ^*$.

#### *Proof*

Let *v* be a node of $G_I$. Let $h_G(v)$ denote its height, i.e., the distance of *v* from the leaves in its subtree. Next, let $X_v$ be the set of all leaves which are descendants of *v* and let $\mu (X_v)$ be the set of leaves of $S_I$ where they map via input mapping $\mu$. Let $x_v$ be the lowest common ancestor of all leaves in $\mu (X_v)$. By $h_S(x_v)$, we will denote the height of node $x_v$ in $S_I$.

Clearly, $\Phi$ must map *v* to $x_v$ or its ancestor, because $\Phi (v)$ must be an ancestor of each leaf in $X_v$. Therefore $\alpha h_G(v)$ must be at least $h_S(x_v)$. We will set $\alpha ^*$ to be the maximum of values $h_S(x_v)/h_G(v)$ for all internal nodes *v* of $G_I$.

Consider now some $\alpha \ge \alpha ^*$ and assume that we use Algorithm 1 for reconciling $\alpha G_I$ to $S_I$. To map an internal node *v* of $G_I$, the algorithm uses an arbitrarily chosen leaf *u* from the subtree rooted at *v*. Since $\alpha h_G(v)\ge h_S(x_v)$, *v* will map to an ancestor of $x_v$. Since $G_I$ and $S_I$ are ultrametric, all choices of *u* will map *v* to the same height, and since this height is at or above the height of the common ancestor $x_v$, all choices of *u* will map *v* to the same node or point of $S_I$. This means that the algorithm will construct a valid isometric reconciliation. □

## Simple histories

All algorithms presented in this paper output inferable histories. An inferable history captures only events that can be inferred from the input partial history. As a result, there is at most one inferable history for each input partial history with rooted species tree. However, it is not always easy to interpret individual nodes of the two trees in an inferable history as evolutionary events (speciations, duplications, losses). Indeed, some nodes may correspond to more than one event and conversely, some events may be missing in the trees. In this section, we define simple histories, in which each node of the species tree corresponds to a single event. These histories thus correspond to an intuitive model of gene family evolution.

Moreover, we describe a correspondence between simple and inferable histories which sheds more light on information missing in the inferable histories returned by our algorithm. Although every inferable history corresponds to some simple history, this simple history may have some branches of zero length. Sometimes we might be interested to know if an inferable history produced by one of our algorithms corresponds to an evolutionary scenario in which only one event happens at any given time on a given branch. This is equivalent to asking if the inferable history corresponds to some simple history with non-zero branch lengths. In Claim 4, we give a characterization of such inferable histories, which can be easily tested algorithmically.

We will start by characterizing nodes of a species tree that correspond to a single evolutionary event. To do so, we need to define an extended inverse mapping $\Phi ^{-1(\mathrm {ext})}(v)$, which contains $\Phi ^{-1}(v)$ as well as $\text{ pt }(a,d(\Phi (a),v))$ for each *a* such that *a* maps to a proper descendant of *v*, and the parent of *a* maps to a proper ancestor of *v* (i.e., if we subdivide edge from *a* to its parent at this point, the new node would also map to *v*). For example in Fig. 3, $\Phi ^{-1(\mathrm {ext})}(x)=\{\text{ pt }(c_1,2),\text{ pt }(c_2,2)\}$.

### **Definition 8**

Consider an arbitrary history $(G, S, \Phi )$ and a node *v* in *S*.

- We call *v* a *sample* if *v* is a leaf (i.e. it corresponds to a sampled taxon).
- We call *v* a *generalized speciation* if it has exactly two children $v_1$ and $v_2$ and each $u \in \Phi ^{-1}(v)$ has exactly two children $u_1$ and $u_2$ such that $\Phi (u_1)$ is a descendant of $v_1$ and $\Phi (u_2)$ is a descendant of $v_2$ (i.e. *v* corresponds to a single speciation, but some genes present in the ancestral species might bypass *v* and continue to one of the daughter species; this is prevented by the next definition).
- We call *v* a *speciation* if it is a generalized speciation and moreover $\Phi ^{-1}(v)=\Phi ^{-1(\mathrm {ext})}(v)$.
- We call *v* a *duplication* if it has one child, $\Phi ^{-1}(v)$ is non-empty and each $u \in \Phi ^{-1}(v)$ has exactly two children.
- We call *v* a *loss* if it has one child, $\Phi ^{-1}(v)$ is non-empty and each $u \in \Phi ^{-1}(v)$ is a leaf in *G*.

Note that a speciation and a sample do not need to have $\Phi ^{-1}$ non-empty, as some species may have lost all copies from the considered family. Also note that a single duplication or deletion event can affect several genes simultaneously, which is consistent with events acting on longer chromosomal segments.

**Fig. 11:**
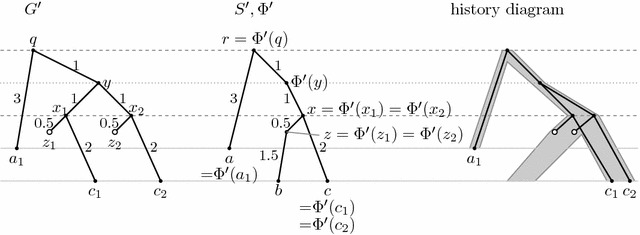
An example of a simple history $(G',S',\Phi ')$. The history $(G,S,\Phi )$ in Fig. 3 is an inferable version of $(G',S',\Phi ')$

For example in tree *S* in Fig. 3, nodes *a*, *b* and *c* are samples, *r* is a speciation, *r* and *x* are generalized speciations and $\Phi (y)$ is a duplication. Node *z* in tree $S'$ in Fig. 11 is a loss.

### **Definition 9**

A *simple history* is a history $(G,S,\Phi )$ in which each node $v\in S$ is either a speciation, a duplication, a loss, or a sample.

### **Definition 10**

Consider a simple history $(G,S,\Phi )$ in which all edges leading to leaves in both trees have positive branch lengths. The *inferable version* of this history is a history obtained as follows.

- Delete each node in *G* with no extant leaf among its descendants.
- Bypass each node in *G* with a single child.
- Bypass each node *v* in *S* if it has a single child and $\Phi ^{-1}(v)$ is empty.
- Contract all edges of length zero in both *G* and *S*.
- If the root *q* of *G* has a single child, delete *q* (the child becomes a new root).
- If the root *r* of *S* has a single child, and $\Phi ^{-1}(r)$ is empty, delete *r* (the child becomes a new root).

For example, history $(G,S,\Phi )$ in Fig. 3 is an inferable version of simple history $(G',S',\Phi ')$ in Fig. 11. The simple history has leaves for deleted genes in the gene tree, and the species tree contains an internal node with one child to which these deleted genes map. Note that the simple history very naturally corresponds to the history diagram shown in these figures.

The following two claims establish relationship between inferable and simple histories.

### **Claim 3**

*Every inferable history is the inferable version of infinitely many simple histories.*

### *Proof*

It is sufficient to prove that every inferable history $(G,S,\Phi )$ corresponds to at least one simple history; additional simple histories can be created by adding unobservable events, such as a duplication followed by a deletion of one of the resulting copies.

Let *v* be a node of *S*. If *v* does not correspond to a single event allowed in simple histories, we will convert it to a series of such events. We will distinguish several cases, depending on the number of children of *v*. If *v* has no children, it is a sample, which is one of allowed node types.

Now let us consider the case when *v* has exactly one child. Note that for a node with one child, $\Phi ^{-1}(v)$ must be non-empty. In addition, each node in $\Phi ^{-1}(v)$ has at least two children because all leaves of *G* are extant, i.e., they map to leaves of *S*, and *G* has no nodes with one child. Let $k\ge 2$ be a value such that each node in $\Phi ^{-1}(v)$ has at most *k* children. We replace node *v* in *S* by a path of nodes $v_1, v_2, \ldots , v_{k-1}$, each having one child. Successive nodes in this path are connected by edges of length zero. The parent of *v* is connected to $v_1$ and the child of *v* is connected to $v_{k-1}$. Each node $u\in \Phi ^{-1}(v)$ with $\ell$ children $(\ell \ge 2$) is similarly replaced by a path of nodes $u_1,\ldots , u_{\ell -1}$ with zero branch lengths. The original children of *u* are distributed among the new nodes so that each new node $u_i$ has exactly two children. Node $u_i$ maps to $v_i$. By this transformation, we have converted *v* to a series of $k-1$ duplications.

Finally, if node *v* from *S* has $g\ge 2$ children, we replace it with $g-1$ speciation nodes $v_1,\ldots , v_{g-1}$ connected by a path with edges of length zero. The original children of *v* are again distributed among these new nodes so that each $v_i$ has exactly two children. We also replace each node $u\in \Phi ^{-1}(v)$ by a path $u_1,\ldots , u_{g-1}$ and map $u_i$ to $v_i$. Each child $u'$ of *u* maps to a descendant of some child $v'$ of *v*. Node $v'$ was placed as a child of some node $v_i$, and similarly, $u'$ will become a child of the corresponding node $u_i$. Ideally, we would have one node $u'$ for every child $v'$ of *v*. If some $v'$ has no corresponding node $u'$, we will create a new leaf $u'$ corresponding to a loss. It will be connected to appropriate $u_i$ by an edge of length smaller than edge from $v_i$ to $v'$. Edge from $v_i$ to $v'$ will be subdivided at the corresponding point so that we can map $u'$. On the other hand, if some $v'$ has multiple corresponding nodes $u'_1, \ldots u'_{\ell }$, we will create a path of $\ell -1$ duplication nodes immediately following $v_i$, similarly as in the case when *v* has one child. Corresponding nodes will be also created in *G* and $u'_1, \ldots u'_{\ell }$ will become their children. Finally, to ensure that $v_1,\ldots , v_{g-1}$ are proper speciation nodes, we will also consider points in $\Phi ^{-1(\mathrm {ext})}(v)$ and subdivide each edge with such a point by a similar path $u_1,\ldots , u_{g-1}$. Since only one branch leading from this path will be non-empty, we will add new leaves for losses similarly as before.

### **Claim 4**

*An inferable history* $(G,S,\Phi )$ *is the inferable version of some simple history with strictly positive branch lengths if and only if every node of* *S* *is a sample, a duplication, or a generalized speciation.*

### *Proof*

If every node of *S* is a sample, a duplication or a generalized speciation, the construction given in the previous claim will provide a history with strictly positive branch lengths.

Now let us assume that $(G,S,\Phi )$ is the inferable version of some simple history $(G', S', \Phi ')$ with positive branch lengths. In the process of obtaining $(G,S,\Phi )$, no edges are contracted, because there are no branches of zero length in $G'$ and $S'$. The only operations performed on $G'$ and $S'$ are deletions of nodes and edges and bypassing nodes with one child. As a result, each node of *G* or *S* corresponds to a single node of $G'$ or $S'$. Also each node of *G* or *S* has at most two children.

Consider node *v* of *S*. If it has no children, it is a sample. If it has one child, $\Phi ^{-1}(v)$ is non-empty and all nodes on $\Phi ^{-1}(v)$ have exactly two children; therefore *v* is a duplication. If *v* has two children in *S*, it must also have two children in $S'$, and thus it is a speciation in $S'$. Each node $u\in \Phi '^{-1}(v)$ thus has two children, each corresponding to a different branch leading from *v*. During changes transforming $(G', S', \Phi ')$ to $(G,S,\Phi )$ some nodes $u\in \Phi '^{-1}(v)$ may be deleted or bypassed, but the remaining ones will satisfy the criteria required by generalized speciation. Thus we have proved that every node of *S* is a sample, a duplication or a generalized speciation.

## Conclusions

In this paper, we have corrected an algorithm for isometric gene tree reconciliation, first presented by Ma et al. [1, 2] in the context of reconstruction of evolutionary histories in the infinite sites model. We have also improved the running time of the algorithm from $O (N^2)$ to $O (N\log N)$, where *N* is the total size of the two input trees.

We have also studied two extensions of the problem. First, we have considered the case when both the gene tree and the species tree are unrooted. We have designed an algorithm with running time $O (N^5\log N)$, which is much slower than the algorithm for reconciling rooted species trees. Perhaps this could be improved by proving a better upper bound on the number of intervals which we need to consider as possible locations of the root. Another related problem is to consider rooted gene tree and unrooted species tree.

In practical applications, we cannot rely on the assumption that the branch lengths are exactly correct. Algorithms that would allow for errors in branch lengths, e.g. assuming that branch lengths are correct up to some degree of tolerance, would be of a large practical value. As the first step in this direction, we have designed an algorithm which allows all branch lengths of the gene tree to be scaled by a constant factor $\alpha$. Such scaling factors are used in practice to model different substitution rates in different gene families. However, we have considered only the case when both input trees are rooted; the variants of the problem with one or both trees unrooted remain open.

## References

[1] Ma J, Ratan A, Raney BJ, Suh BB, Miller W, Haussler D (2008). The infinite sites model of genome evolution. Proc Natl Acad Sci.

[2] Ma J, Ratan A, Raney BJ, Suh BB, Zhang L, Miller W, Haussler D (2008). DUPCAR: reconstructing contiguous ancestral regions with duplications. J Comput Biol.

[3] Felsenstein J (2004). Inferring phylogenies.

[4] Goodman M, Czelusniak J, Moore GW, Romero-Herrera A, Matsuda G (1979). Fitting the gene lineage into its species lineage, a parsimony strategy illustrated by cladograms constructed from globin sequences. Syst Biol.

[5] Guigo R, Muchnik I, Smith TF (1996). Reconstruction of ancient molecular phylogeny. Mol Phylogenet Evol.

[6] Zhang L (1997). On a Mirkin–Muchnik–Smith conjecture for comparing molecular phylogenies. J Comput Biol.

[7] Eulenstein O. A linear time algorithm for tree mapping. GMD-Forschungszentrum Informationstechnik (1997).

[8] Zmasek CM, Eddy SR (2001). A simple algorithm to infer gene duplication and speciation events on a gene tree. Bioinformatics.

[9] Fertin G, Labarre A, Rusu I, Tannier E, Vialette S (2009). Combinatorics of genome rearrangements.

[10] Sennblad B, Lagergren J (2009). Probabilistic orthology analysis. Syst Biol.

[11] Górecki P, Burleigh GJ, Eulenstein O (2011). Maximum likelihood models and algorithms for gene tree evolution with duplications and losses. BMC Bioinform.

[12] Doyon J-P, Hamel S, Chauve C (2012). An efficient method for exploring the space of gene tree/species tree reconciliations in a probabilistic framework. IEEE/ACM Trans Comput Biol Bioinfor.

[13] Doyon JP, Scornavacca C, Gorbunov KY, Szöllősi GJ, Ranwez V, Berry V. An efficient algorithm for gene/species trees parsimonious reconciliation with losses, duplications and transfers. In: Comparative genomics (RECOMB-CG 2012) vol. 6398, pp. 93–108. Berlin: Springer; 2010.

[14] Bansal MS, Alm EJ, Kellis M (2012). Efficient algorithms for the reconciliation problem with gene duplication, horizontal transfer and loss. Bioinformatics.

[15] Harel D, Tarjan RE (1984). Fast algorithms for finding nearest common ancestors. SIAM J Comput.

[16] Bender MA, Farach-Colton M. The LCA problem revisited. In: LATIN 2000: theoretical informatics, vol. 1776, pp. 88–94. Berlin: Springer; 2000.

[17] Berkman O, Vishkin U (1994). Finding level-ancestors in trees. J Comput Syst Sci.

[18] Amir A, Landau GM, Lewenstein M, Sokol D (2007). Dynamic text and static pattern matching. ACM Trans Algorithm.

